# Antioxidant Properties of *Berberis aetnensis* C. Presl (Berberidaceae) Roots Extract and Protective Effects on Astroglial Cell Cultures

**DOI:** 10.1155/2014/315473

**Published:** 2014-08-11

**Authors:** Agata Campisi, Rosaria Acquaviva, Roberta Bonfanti, Giuseppina Raciti, Andrea Amodeo, Silvana Mastrojeni, Salvatore Ragusa, Liliana Iauk

**Affiliations:** ^1^Department of Drug Science, Biochemistry Section, University of Catania, Viale Andrea Doria 6, 95123 Catania, Italy; ^2^Department of Biomedical Sciences, Microbiology Section, University of Catania, Via Androne 81, 95125 Catania, Italy; ^3^Department of Health Sciences, University “Magna Graecia” of Catanzaro, Viale Europa, 88100 Catanzaro, Italy

## Abstract

*Berberis aetnensis* C. Presl (Berberidaceae) is a bushy-spiny shrub common on Mount Etna (Sicily). We demonstrated that the alkaloid extract of roots of *B. aetnensis* C. Presl contains prevalently berberine and berbamine, possesses antimicrobial properties, and was able to counteract the upregulation evoked by glutamate of tissue transglutaminase in primary rat astroglial cell cultures. Until now, there are no reports regarding antioxidant properties of *B. aetnensis* C. Presl collected in Sicily. Air-dried, powdered roots of *B. aetnensis* C. Presl were extracted, identified, and quantified by HPLC. We assessed in cellular free system its effect on superoxide anion, radicals scavenging activity of antioxidants against free radicals like the 1,1-diphenyl-2-picrylhydrazyl radical, and the inhibition of xanthine oxidase activity. In primary rat astroglial cell cultures, exposed to glutamate, we evaluated the effect of the extract on glutathione levels and on intracellular production of reactive oxygen species generated by glutamate. The alkaloid extract of *B. aetnensis* C. Presl inhibited superoxide anion, restored to control values, the decrease of GSH levels, and the production of reactive oxygen species. Potent antioxidant activities of the alkaloid extract of roots of *B. aetnensis* C. Presl may be one of the mechanisms by which the extract is effective against health disorders associated to oxidative stress.

## 1. Introduction

*Berberis aetnensis* C. Presl (Berberidaceae) is a bushy-spiny shrub with dense twisted branches whose grey, smooth bark has fine longitudinal channels. Leaves on long branches are transformed into spines 3-fid, and the shorter branches have normal leaves. The leaves are oblong-egg shaped, rigid, and coriaceous and have spiny-serrated margins. The flowers are yellow in colour and are grouped in hanging racemes. The fruit is an oblong berry and dark red in colour. As soon as they are cut, the branches and the roots have a penetrating odour and are intensely yellow in colour.* B. aetnensis* C. Presl grows on rocky slopes, on the rocky bed of water courses, at the upper limit of the wooded zone, and beyond. In Sicily it is common on Mount Etna, in the area of vegetation between 1800 and 2400–2500 m [1, 2].

In previous research, we showed that the major compound in* B. aetnensis* C. Presl characterized by HPLC analysis is berberine [3–5]. It also contains smaller amounts of berbamine and jatrorrhizine and even lesser amounts of palmatine, oxyacanthine, and other alkaloids [5]. We also demonstrated that the extract possesses antimicrobial properties [3] and that it was able to counteract the upregulation evoked by glutamate of tissue transglutaminase (TG2) in primary rat astroglial cell cultures [5]. However, until now, there are no reports regarding its antioxidant properties. In particular we have reported only the antioxidant activity of* B. aetnensis* C. Presl collected in Calabria [6]. It is known that the climatic conditions and habitat changes induce modifications in the growth and metabolism of plants and of their active compounds. Several studies reported that oxidative stress has been associated with pathological conditions, including central nervous system diseases, such as Parkinson's syndrome, Alzheimer's disease (AD), cerebral ischemia, and physiological brain aging processes [7].

The formation and release of radical oxygen reactive species (ROS) cause structural and functional alterations of cell membranes. Free radicals attack polyunsaturated fatty acids in biomembranes and mitochondria begin the main source of ROS, when the mitochondrial respiratory chain is impaired. In these cases, a compound possessing antioxidant properties can be useful in stopping ROS production and limiting oxidative cell damages [8, 9].

Experience from ethnomedicine, together with extensive basic laboratory findings, has shown for many years that flavonoids and alkaloid compounds could play an important role in prevention and treatment of oxidative stress.

Herein, we assessed the antioxidant activity of the alkaloid extract of roots of* B. aetnensis* C. Presl both in an* in vitro* cellular free system and an* in vitro* cellular system. In particular, we evaluated* in vitro* cellular free system, the effect of the alkaloid extract on superoxide anion, the radicals scavenging activity of antioxidants against free radicals like the 1,1-diphenyl-2-picrylhydrazyl (DPPH) radical, and the inhibition of xanthine oxidase (XO) activity. In an* in vitro* cellular system, we also assessed total glutathione intracellular content (GSH + GSSG) and the intracellular production of ROS.

## 2. Materials and Methods

### 2.1. Chemicals

1,1-Diphenyl-2-picrylhydrazyl (DPPH) radical, 2′,7′-dichlorofluorescein diacetate (DCFH-DA), Trolox, Allopurinol, superoxide dismutase (SOD), NADH, and other analytical chemicals were purchased from Sigma-Aldrich Chimica Srl (Milan, Italy). Dulbecco's modified Eagle's medium (DMEM) and heat-inactivated fetal bovine serum (FBS) were obtained from Invitrogen (Milan, Italy). Mouse monoclonal antibody against glial fibrillary acidic protein (GFAP) was from Chemicon (Prodotti Gianni, Milan, Italy).

#### 2.1.1. Plant Collection and Preparation of Extracts

The roots of* B. aetnensis* C. Presl were collected on the slopes of the volcano Mount Etna (1800–1900 m) thanks to the Commandant of the Regional Forest Corps Detachment of Catania-Nicolosi (Sicily, Italy), Warrant Officer Gianluca Ferlito. A voucher specimen of the plant number 18/03 was deposited in the herbarium of Department of Health Sciences, University “Magna Graecia” of Catanzaro (Italy).

The air-dried, powdered roots of* B. aetnensis* C. Presl were extracted with methanol five times by maceration. Solution was evaporated to dryness to give a total extract. This extract was suspended in methanol and partitioned with* n*-hexane to yield an* n*-hexane fraction. Then, the methanol fraction was dissolved in HCl 10%, and the yellow coloured precipitate thus occurred was filtered. The precipitate was purified by crystallisation from ethanol and was found to possess berberine. A solution of NH_4_OH 25% was added to the remaining acidified extract to adjust pH 8–10 and then extracted with dichloromethane. The combined dichloromethane solutions were evaporated to dryness under reduced pressure to yield an alkaloid fraction.

#### 2.1.2. Determination and Identification of Alkaloids by High-Performance Liquid Chromatography (HPLC)

Gradient-elution HPLC of the alkaloid residue and reference compounds (berberine, berbamine, palmatine, jatrorrhizine, and oxyacanthine) was carried out on a Hewlett Packard liquid chromatograph (HPLC 1100) equipped with a quaternary solvent pump, an autosampler, a thermostat, and a photodiode array detector. A Spherisorb S5ODS1 reversed-phase column (250 × 4.6 mm i.d. with 5.0 mm particle size) with a ODS guard-column (4 × 20 mm i.d.) was used [10, 11].

#### 2.1.3. Determination of Antioxidant Activity* In Vitro* Cellular Free System

*Scavenger Effect on Superoxide Anion.* Superoxide anion was generated* in vitro* as reported by Acquaviva et al. [12]. The assay mixture contained in a total volume of 1 mL, 100 mM triethanolamine-diethanolamine buffer, pH 7.4, 3 mM NADH, 25 mM, 12.5 mM EDTA/MnCl_2_, and 10 mM *β*-mercaptoethanol; some samples contained the alkaloid extract of* B. aetnensis* C. Presl roots at different concentrations (0.05–2 *μ*g/mL). After 20 min of incubation at 25°C, the decrease in absorbance was measured at 340 nm. SOD (80 mU/mL) as a standard was used.

*Quenching of DPPH Radical*. The reaction mixture contained 86 *μ*M DPPH radical and the alkaloid extract of* B. aetnensis* C. Presl roots at different concentrations (12.5–200 *μ*g/mL) in 1 mL of ethanol. After 10 min at room temperature the absorbance at 517 nm was recorded [12]. Trolox (30 *μ*M), water-soluble derivative of vitamin E, as a standard was used.

*Xanthine Oxidase Activity Inhibition*. Xanthine oxidase activity (Xanthine: oxygen oxidoreductase, E.C.1.1.3.22, XO) was evaluated spectrophotometrically by following the formation of uric acid at 292 nm (*ε*_*M*_ = 9.2 × 10^3^) [12]. The assay mixture contained, in a final volume of 1 mL, 50 mM phosphate buffer pH 7.8, 25 *μ*M solution of xanthine and 24 mU XO (specific activity 1 U/mg of protein), and different concentrations of the alkaloid extract of* B. aetnensis* C. Presl roots (25–100 *μ*g/mL). Allopurinol (30 *μ*M), a competitive inhibitor of XO, as a standard was used. The results were expressed as percentage of inhibition of the enzyme activity.

#### 2.1.4. Determination of Antioxidant Activity* In Vitro* Cellular System

*Cell Cultures*. Primary cultures of astrocytes were prepared from newborn albino rat brains (from 1- to 2-day-old Wistar strain rats) as described [13]. Briefly, cerebral tissues, after dissection and careful removal of the meninges, were mechanically dissociated through 82 *μ*m pore sterile mesh (Nitex). Isolated cells were suspended in DMEM, supplemented with 20% (v/v) FBS, 2 mM glutamine, streptomycin (50 mg/mL), and penicillin (50 U/mL) and plated at a density of 3 × 10^6^ cells/100 mm dishes and of 0.5–10^5^ cells/chamber of multichambered slides. Cells were maintained at 37°C in a 5% CO_2_/95% air humidified atmosphere for two weeks and medium exchanged every three days. The low initial plating density of dissociated cells was meant to favour the growth of astrocytes with only a very little oligodendroglial and microglial cells contamination. Astroglial cell cultures were characterized at 14 days* in vitro* (DIV), which is confluent, by immunofluorescent staining with GFAP [13]. All experiments conformed to the guidelines of the Ethical Committee of the University of Catania, Italy, and were carried out in accordance with EC Directive 86/609/EEC for animal experiments.

*Treatment of the Cells*. Astrocytes at 14 DIV were treated with glutamate (500 *μ*M) for 24 h as previously described [14]. Other cultures were treated with different concentrations of the alkaloid extract of* B. aetnensis* C. Presl roots (5–20 *μ*g/mL), which were added 20 min before glutamate exposure.

*Glutathione Measurement*. Astroglial cell cultures were scraped off and lysed in 50 *μ*M sodium phosphate buffer, pH 7.4. The protein concentration in cell extracts was determined by Bradford assay [15]. Then, total glutathione intracellular content (GSH + GSSG) was chemically determined as described by Chen et al. [16], using a Hitachi U-2000 spectrophotometer (Hitachi, Tokyo, Japan).

*ROS Levels Determination*. Reactive species determination was performed by using DCFH-DA as a fluorescent probe. Briefly, 100 *μ*M of DCHF-DA was dissolved in 100% methanol and added to the cellular medium, and cells were incubated at 37°C for 30 minutes. Under these conditions, the acetate group was not hydrolyzed [17]. After incubation, astrocytes were lysed and centrifuged at 10,000 ×g for 10 minutes. The fluorescence (corresponding to the radical-species-oxidized 2′,7′-dichlorofluorescein; DCF) was monitored spectrofluorometrically (excitation, *λ* = 488 nm; emission, *λ* = 525 nm), using an F-2000 spectrofluorometer (Hitachi). Values are expressed as fluorescence intensity/mg protein. Protein concentration was measured, according to Bradford [15].

### 2.2. Statistical Analysis

All values are presented as means ± SD of five separate experiments. Statistical analysis was performed using one-way ANOVA, followed by Newman-Keuls post-hoc test. Differences were considered statistically significant at ^a^ *P* < 0.05 and ^b^ *P* < 0.001.

## 3. Results and Discussion

In this study we assessed the antioxidant activity of the alkaloid extract of roots of* B. aetnensis* C. Presl both in an* in vitro* cellular free system and an* in vitro* cellular model.

Oxidative stress is the causative agent in a number of human diseases, such as atherosclerosis, ischemic reperfusion injury, inflammation, carcinogenesis, ageing, and neurodegenerative diseases (Parkinson and Alzheimer). Although there are many determinants in the development of these diseases, considerable experimental evidence links the production of ROS to biological damage that can potentially provide a mechanistic basis for their initiation and/or progression [18–22].

In recent years, there has been great interest in the health effects of various herbal and in the* in vivo* protective function of natural antioxidants contained in dietary plants against oxidative damage caused by ROS [23, 24].

The vast structural diversity of natural compounds found in planta provides unique opportunities for discovering new drugs that rationally target the abnormal molecular and biochemical signals leading to oxidative diseases.

It is well known that Berberis plant extracts have been involved in a whole host of biological responses, acting, for example, as anti-inflammatory, rheumatic, hypotensive, febrifugal, analgesic, muscle relaxant, and antihyperglycemic agents among others. Since the antioxidant effects of natural compounds could be mainly due to their free radical scavenging and/or chelating activities, in this study the direct superoxide anion scavenging capacity of this natural product was investigated using a method which excludes the Fenton-type reaction and xanthine/xanthine oxidase system [25, 26].

**Figure 1:**
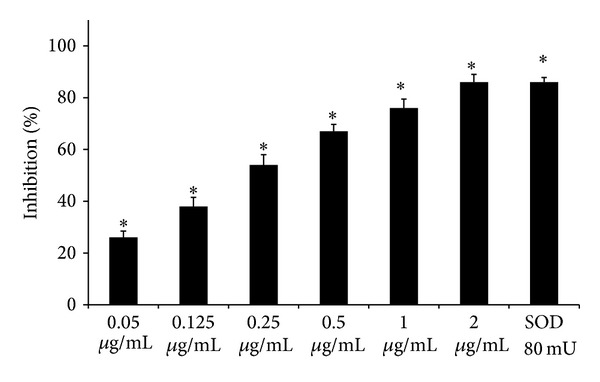
Scavenger effect of* B. aetnensis* C. Presl chloroformic extract and superoxide dismutase (SOD) activity on O_2_^•−^ expressed as percentage of inhibition of NADH oxidation. Rate of O_2_^•−^ production was expressed as nmoles of O_2_^•−^ produced/min. Each value represents the mean ± SD of 5 separate experiments. Significance versus control (sample without extract) *P < 0.001.

In Figure 1 we showed that the alkaloid extract of* B. aetnensis* C. Presl was able to inhibit superoxide anion formation in a dose-dependent manner. Its effect was compared with SOD activity. We found that the extract at 2 *μ*g/mL concentration possessed a similar action of 30 mU of SOD activity.

The free radical scavenging activity of extract of* B. aetnensis* C. Presl was also tested measuring its ability to bleach the stable DPPH radical. These analytical methods measure the radicals scavenging activity of antioxidants against free radicals like the DPPH radical, the superoxide anion radical (O_2_^•−^), the hydroxyl radical (^•^OH), or the peroxyl radical (ROO^•^).

**Figure 2:**
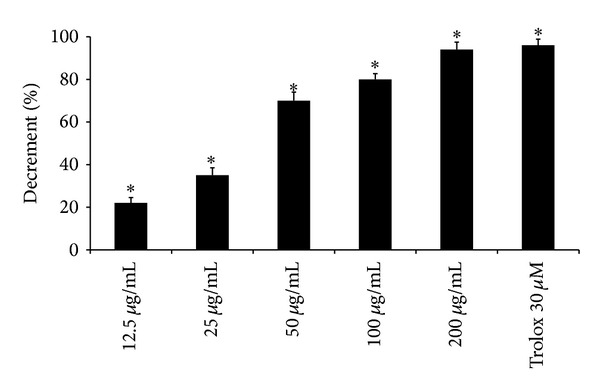
Scavenger effect of* B*.* aetnensis* C. Presl chloroformic extract and Trolox expressed as capacity to bleach DPPH. Results are expressed as percentage of the decrease in absorbance at *λ* = 517 nm when compared with the control. Each value represents the mean ± SD of 5 separate experiments. Significance versus control *P < 0.001.

**Figure 3:**
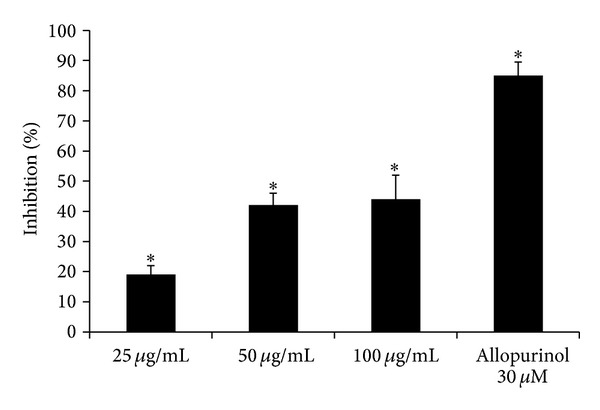
Effect of* B*.* aetnensis* C. Presl chloroformic extract on xanthine oxidase activity. Results are expressed as percentage of inhibition of XO activity when compared with the control. Each value represents the mean ± SD of 5 separate experiments. Significance versus control (sample without extract) *P < 0.001.

Our results demonstrate that the alkaloid extract of roots of* B. aetnensis* C. Presl presented a DPPH quenching capacity in a dose dependent manner (Figure 2). This effect was compared with Trolox, a water-soluble vitamin E analogue. Its effect, at 200 *μ*g/mL concentration, was equivalent to 30 *μ*M of Trolox (Figure 2). These data are in agreement with a recent study demonstrating that* B. aetnensis* C. Presl, collected in Calabria, has activity against DPPH tests [6]. The concentrations of the extract of* B. aetnensis* C. Presl in the DPPH test are higher than those used to evaluate the scavenger effect on superoxide anion probably to the large size and high stability of DPPH radical with respect to O_2_^•−^. Since XO activity represents a physiological source of superoxide anions in eukaryotic cells, we assessed if the extract might inhibit the enzyme activity. We observed that the alkaloid extract of* B. aetnensis* C. Presl was able to inhibit, in a dose-dependent manner (25, 50, 100 *μ*g/mL), XO activity. The effect of the extract was compared with Allopurinol, a specific competitive inhibitor of XO used as a standard (Figure 3). We found that its effect was lower than 30 *μ*M Allopurinol (Figure 3). Also in this test, the concentrations of the extract of* B. aetnensis* C. Presl are superior because this test reproduces a physiological enzyme system.

Our results demonstrate that* B. aetnensis* C. Presl extract was able to inhibit superoxide anion generation in a dose-dependent manner, indicating also that this extract could be bleached by the nonenzymatic reaction with the superoxide radical and no as a chelating agent. The inhibitory action of* B. aetnensis* C. Presl on O_2_^•−^ production in the xanthine-xanthine oxidase system may be due either to scavenger activity of these compounds or to their action on the primary function of the enzyme. Furthermore, our findings could explain the protective effects of the species of* Berberis aetnensis* C. Presl on those diseases which are characterized by an overproduction of free radicals, in particular of the O_2_^•−^, due to both electron leak in the mitochondrial respiratory chain and the conversion of xanthine dehydrogenase to XO producing O_2_^•−^ when it oxidizes xanthine to uric acid [27].

The antioxidant effect of the alkaloid extract of* B. aetnensis* C. Presl roots was also assessed in cellular system using primary rat which exposed the astroglial cell cultures to 500 *μ*M glutamate for 24 h.

Cells were characterized by immunofluorescent staining used as marker GFAP [13]. About 90% of cells were GFAP positive, indicating that cultures were highly enriched in astrocytes (data not shown).

**Figure 4:**
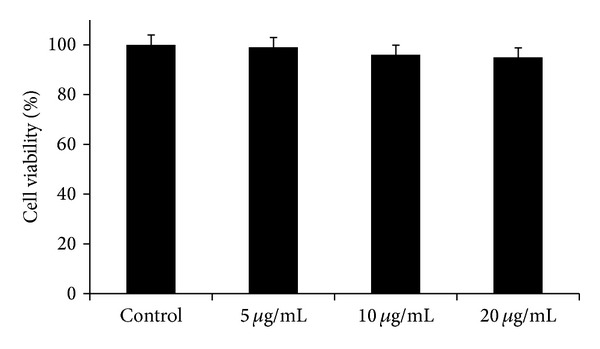
Cell viability in rat astrocytes after treatment with different concentrations* B. aetnensis* C. Presl. Each value represents the mean ± SD of 5 separate experiments.

In preliminary experiments, on the basis of both microscopy observations and MTT assay, we established the nontoxic concentration of the extract for astroglial cell cultures. The alkaloid extract of* B. aetnensis* C. Presl (5, 10, 20 *μ*g/mL) both in absence or in presence of 500 *μ*M glutamate for 24 h did not modify cellular vitality of the cultures (Figure 4).

We used stressor glutamate because its high levels induce alterations in glutamate transport, mitochondria impairment, decrease in ATP levels, GSH depletion, ROS production, macromolecular synthesis, and subsequent neuronal cell death [28–30].

The glutamate-evoked oxidative stress was evaluated by measuring the depletion of intracellular GSH levels and ROS production. Glutamate produced a significant decrease in the intracellular GSH levels and a significant increase of ROS levels, when compared to the untreated control (Tables 1 and 2). Alkaloid extract of* B. aetnensis* C. Presl alone (5–10–20 *μ*g/mL) did not modify GSH and ROS levels when compared to the untreated control. The preincubation of the cultures with the extract of* B. aetnensis* C. Presl (5, 10, 20 *μ*g/mL) was able to counteract the effect of glutamate restoring, in a dose-dependent manner, GSH and ROS levels (Tables 1 and 2). In particular, 20 *μ*g/mL of the extract showed values similar to untreated control (Tables 1 and 2).

The protective effect against glutamate toxicity of the alkaloid extract of* B. aetnensis* C. Presl roots appeared stronger than that of the synthetic antioxidant compounds used in our previous research [5].

This extract, which mostly contains berberine, might be able to counteract the excitotoxicity induced by glutamate exposure which leads to an increase in intracellular Ca^++^ levels, ROS increase production, and depletion of GSH levels. Consequently, several calcium-dependent enzymes may be activated, causing mitochondria impairment, decrease in ATP levels, and subsequent neuronal cell death [31]. Berberine and the alkaloid extract of* B. aetnensis* C. Presl might be also active because it reduces the overexpression of TG2 induced by treatment with glutamate [5]. Increased formation of ROS may be antagonized by endogenous and exogenous antioxidants; then this extract might change not only the state intracellular oxidative status modified by oxidative stress but also increase antioxidant defences.

The antioxidant effect of the extract might be related to its elevated levels of berberine, which also represents the main alkaloid in all plants of the genus Berberis, which has potent neuroprotective effects against ischemic damage in cerebral ischemia/reperfusion models [11, 32, 33]. In addition, it potentiates the nerve growth factor- (NGF) induced differentiation in neural cells [34].

Thus, these findings, the alkaloid extract of* B. aetnensis* C. Presl roots, possess antioxidant properties, which are probably prevalent due to the higher content of berberine as reported in our previous study [5].

HPLC analysis showed that the major compound present in the extract was berberine (376 mg/g). It also contains smaller amounts of berbamine (35 mg/g) and jatrorrhizine (33 mg/g) and even lesser amounts of palmatine (12 mg/g), oxyacanthine (9 mg/g), and other alkaloids.

Our results are in agreement with other reports on the alkaloids from the roots of* Berberis* species [35–38]. Furthermore, it is possible to assume that the alkaloid extract of* B. aetnensis* C. Presl roots is able to counteract glutamate uptake-induced impairment of cystine/glutamate antiporter which leads to depletion of GSH content and biochemical alterations, resulting in the delayed toxic effect for primary astrocyte cultures [28, 37]. Then dietary supplementation of* B. aetnensis* C. Presl roots extract to quails reduces the detrimental effects of oxidative stress. In conclusion, our results could explain the protective effects of* B. aetnensis* C. Presl on those diseases which are characterized by an overproduction of free radicals, in particular of the superoxide anion, and suggest that the alkaloid extract of* B. aetnensis* C. Presl roots, which mostly contains berberine, ameliorating the excessive production of glutamate, may represent a natural therapeutic strategy in the neuropathological conditions associated with excitotoxicity. However, other studies are now in progress to better clarify the molecular mechanisms of the alkaloid extract of* B. aetnensis* C. Presl roots.

## Figures and Tables

**Table 1:** Effect of *B. aetnensis* C. Presl chloroformic extract on GSH levels in primary rat astroglial cell cultures at 14 DIV exposed to 500 *μ*M glutamate for 24 hrs.

Treatment	nmoles/mg protein
Control	15 ± 0.9
5 *μ*g/mL *B. aetnensis* C. Presl	15 ± 0.2
10 *μ*g/mL *B. aetnensis* C. Presl	14.8 ± 0.6
20 *μ*g/mL *B. aetnensis* C. Presl	14.7 ± 0.9
Glutamate 500 *μ*M	9.5 ± 0.4^a^
Glutamate 500 *μ*M + 5 *μ*g/mL *B. aetnensis* C. Presl	10.6 ± 0.3^a^
Glutamate 500 *μ*M + 10 *μ*g/mL *B. aetnensis* C. Presl	11.20 ± 0.5^ab^
Glutamate 500 *μ*M + 20 *μ*g/mL *B. aetnensis* C. Presl	14 ± 0.3^b^

GSH levels are expressed as nmol of GSH/mg protein. Each value represents the mean ± SD of 5 separate experiments. ^a^ *P* < 0.01 versus control and ^b^ *P* < 0.01 versus glutamate.

**Table 2:** Effect of *B. aetnensis* C. Presl chloroformic extract on ROS levels in primary rat astroglial cell cultures at 14 DIV exposed to 500 *μ*M glutamate for 24 hrs.

Treatment	I.F./mg protein
Control	3.8 ± 0.52
5 *μ*g/mL *B. aetnensis* C. Presl	3.75 ± 0.6
10 *μ*g/mL *B. aetnensis* C. Presl	3.81 ± 0.45
20 *μ*g/mL *B. aetnensis* C. Presl	3.78 ± 0.55
Glutamate 500 *μ*M	6.8 ± 0.9^a^
Glutamate 500 *μ*M + 5 *μ*g/mL *B. aetnensis* C. Presl	5.2 ± 0.5^ab^
Glutamate 500 *μ*M + 10 *μ*g/mL *B. aetnensis* C. Presl	4.6 ± 0.7^ab^
Glutamate 500 *μ*M + 20 *μ*g/mL *B. aetnensis* C. Presl	4 ± 0.5^b^

ROS levels are expressed as nmol of dichlorofluorescein produced 30 min/mg protein. Each value represents the mean ± SD of 5 separate experiments. ^a^ *P* < 0.05 versus control and ^b^ *P* < 0.05 versus glutamate.
